# Effects of bariatric surgery on gout incidence in the Swedish Obese Subjects study: a non-randomised, prospective, controlled intervention trial

**DOI:** 10.1136/annrheumdis-2016-209958

**Published:** 2016-10-08

**Authors:** Cristina Maglio, Markku Peltonen, Martin Neovius, Peter Jacobson, Lennart Jacobsson, Anna Rudin, Lena M S Carlsson

**Affiliations:** 1 Department of Molecular and Clinical Medicine, Institute of Medicine, The Sahlgrenska Academy at University of Gothenburg, Gothenburg, Sweden; 2 Department of Rheumatology and Inflammation Research, Institute of Medicine, The Sahlgrenska Academy at University of Gothenburg, Gothenburg, Sweden; 3 Department of Chronic Disease Prevention, National Institute for Health and Welfare, Helsinki, Finland; 4 Clinical Epidemiology Unit, Department of Medicine, Karolinska Institutet, Stockholm, Sweden

**Keywords:** Gout, Arthritis, Treatment

## Abstract

**Objectives:**

To assess the long-term effect of bariatric surgery on the incidence of gout and hyperuricaemia in participants of the Swedish Obese Subjects (SOS) study.

**Methods:**

This report includes 1982 subjects who underwent bariatric surgery and 1999 obese controls from the SOS study, a prospective intervention trial designed to assess the effect of bariatric surgery compared with conventional treatment. None of the subjects had gout at baseline. An endpoint on gout incidence was created based on information on gout diagnosis and use of gout medications through national registers and questionnaires. Median follow-up for the incidence of gout was about 19 years for both groups. Moreover, the incidence of hyperuricaemia over up to 20 years was examined in a subgroup of participants having baseline uric acid levels <6.8 mg/dL.

**Results:**

Bariatric surgery was associated with a reduced incidence of gout compared with usual care (adjusted HR 0.60, 95% CI 0.48 to 0.75, p<0.001). The difference in absolute risk between groups was 3 percentage points at 15 years, and the number of subjects needed to be treated by bariatric surgery to prevent one incident gout event was 32 (95% CI 22 to 59). The effect of bariatric surgery on gout incidence was not influenced by baseline risk factors, including body mass index. During follow-up, the surgery group had a lower incidence of hyperuricaemia (adjusted HR 0.47, 95% CI 0.39 to 0.58, p<0.001). The difference in absolute risk between groups was 12 percentage points at 15 years, and the number of participants needed to be treated by bariatric surgery to prevent hyperuricaemia was 8 (95% CI 6 to 13).

**Conclusions:**

Bariatric surgery prevents gout and hyperuricaemia in obese subjects.

**Trial registration number:**

NCT01479452; Results.

## Introduction

Gout is a common type of arthritis characterised by recurrent acute episodes of inflammation in joints or tendons.^1^ Gout is caused by the deposition of monosodium urate crystals^2^ ^3^ which occurs when serum uric acid levels exceed the saturation threshold which is 6.8 mg/dL (around 405 µmol/L) at physiological temperature.^4^ However, only a small percentage of subjects with increased serum uric acid levels develop gout.^5^ Additional factors are therefore likely to contribute to the disease pathogenesis.^6^ ^7^ The prevalence of gout is increasing worldwide, and obesity is one of the most common risk factors for gout development.^8^ Weight loss is associated with a reduction in serum urate levels^9–11^ and with a lower incidence of gouty arthritis.^11^ ^12^ The American College of Rheumatology and the European League against Rheumatism guidelines recommend weight loss for gout management in obese subjects.^13^ ^14^

Bariatric surgery is the most effective means of achieving substantial and sustained weight loss in obese subjects.^15^ ^16^ The beneficial effects of bariatric surgery are not limited to weight loss, but they extend to improvement in metabolic parameters^17^ and to lower risk of developing type 2 diabetes, cardiovascular disease and cancer.^18–23^ Bariatric surgery leads also to lower serum uric acid levels,^24–26^ and a recent study suggests that it reduces the incidence of gouty attacks up to 1-year follow-up in subjects having a previous gout diagnosis.^27^

The Swedish Obese Subjects (SOS) study is a non-randomised, controlled, prospective intervention trial designed to assess the effect of bariatric surgery on obesity-associated morbidity and mortality, compared with conventional treatment.^15^ ^28^ In a previous report on cardiovascular risk factors, we showed that bariatric surgery reduces serum uric acid levels up to 10 years after treatment.^17^ The aim of the current analysis was to assess the long-term effect of bariatric surgery on the incidence of gout and hyperuricaemia in subjects without a previous gout diagnosis.

## Methods

### SOS study design

The ongoing SOS intervention trial recruited 4047 obese subjects from 25 Swedish surgical departments and 480 Swedish primary healthcare centres between September 1987 and January 2001, as previously reported.^15^ Of these, 2010 subjects chose to undergo bariatric surgery (surgery group), and 2037 subjects formed the control group. The control group was created using 18 matching variables (ie, sex, age, weight, height, waist circumference, hip circumference, systolic blood pressure, serum total cholesterol, serum triglycerides, smoking, diabetes, menopausal status, four psychosocial variables associated with death risk and two personality traits related to treatment preferences). The matching was not performed at an individual level, although the surgery patient and the matched control patient started the study on the same day (ie, the day of the surgery). Instead, controls were selected using a matching algorithm, so that the current mean values of the matching variables in the control group became as similar as possible to the current mean values in the surgery group according to the method of sequential treatment assignment.^29^ Inclusion criteria, which were identical for both groups, were age 37–60 years and body mass index (weight in kilograms divided by the square of the height in metres) ≥34 for men and ≥38 for women. Exclusion criteria were earlier surgery for gastric or duodenal ulcer, earlier bariatric surgery, gastric ulcer during the past 6 months, ongoing or active malignancy during the past 5 years, myocardial infarction during the past 6 months, bulimic eating pattern, drug or alcohol abuse, psychiatric or cooperative problems contraindicating bariatric surgery and other rare contraindicating conditions.

In the surgery group, 376 subjects underwent gastric banding, 1369 underwent vertical banded gastroplasty and 265 underwent gastric bypass. The control group received conventional non-surgical obesity treatment at their centres of registration, ranging from advanced lifestyle modification (including recommendations regarding eating behaviour, food selection, energy intake and physical activity) to no treatment whatsoever. Physical examinations were performed at matching, at baseline and after 6 months and 1, 2, 3, 4, 6, 8, 10, 15 and 20 years. At the same time points, questionnaires including a question about current drug consumption were collected. Centralised laboratory examinations, including measurement of serum uric acid levels, were performed at matching, at baseline and after 2, 10, 15 and 20 years.

The primary endpoint of the SOS study was mortality.^28^ Secondary endpoints included type 2 diabetes and cardiovascular disease. Gout incidence was not a predefined endpoint. Type 2 diabetes was defined as fasting blood glucose ≥110 mg/dL and/or self-reported therapy with glucose-lowering medications. Hypertension was defined as systolic blood pressure ≥140 mm Hg or diastolic blood pressure ≥90 mm Hg or treatment with antihypertensive medication. Previous cardiovascular events were defined as myocardial infarction or cerebral stroke before inclusion in the study.

Written or oral informed consent was obtained from all the study subjects. Seven local ethics review boards approved the SOS study protocol. The study has been registered at ClinicalTrials.gov with identifier NCT01479452.

### Gout and hyperuricaemia

The current report includes 1982 subjects who underwent bariatric surgery and 1999 controls, none of whom had gout at baseline. In the surgery group, 372 subjects underwent gastric banding, 1347 underwent vertical banded gastroplasty and 263 underwent gastric bypass. According to questionnaires administered to study participants at 6 months, 1 year and 2 years, 1009 subjects (50.5%) from the control group had tried to lose weight with professional guidance, whereas 141 (7.1%) participants used the anti-obesity drugs orlistat, sibutramine or rimonabant at least once. A total of 284 participants (14%) from the control group underwent bariatric surgery during follow-up and have been excluded by the per-protocol analysis.

A composite endpoint of gout incidence during follow-up was created based on the information on gout diagnosis and the use of gout medications (see online supplementary figure S1A). Data on gout diagnosis were obtained from the National Patient Register which includes medical records from inpatient and non-primary care outpatient visits. Inpatient care has been recorded since 1964 and reached national coverage in 1987. Recording of non-primary outpatient care, which includes visits to hospital-based medical specialists, was started nationwide in 2001. The National Patient Register was searched for the following International Classification of Diseases (ICD) codes for gout: 274 (ICD-8); 274 and 712 (ICD-9); M10–11 (ICD-10). The Prescribed Drug Register, which includes information on all dispensed prescription drugs in Sweden from July 2005, was searched for the gout medications allopurinol (Anatomical Therapeutic Chemical code: M04AA01) and colchicine (M04AC01); we chose not to include probenecid, since its indication for gout treatment is not unequivocal. In addition, self-reported data on consumption of allopurinol and colchicine were collected from the patients' questionnaires at every visit. In total, 339 gout events occurred during follow-up, of which 279 were detected according to self-reported consumption of gout drugs or the Prescribed Drug Register, whereas 60 were detected through the National Patient Register. The patients' questionnaires were also used to obtain self-reported baseline consumption of medications associated with gout risk (ie, diuretics, β-blockers, ACE inhibitors, angiotensin II receptor blockers, low-dose acetylsalicylic acid, statins, tacrolimus, ciclosporin and organic nitrates^30–32^). However, no consumption of ACE inhibitors, angiotensin II receptor blockers or tacrolimus was reported at baseline.

10.1136/annrheumdis-2016-209958.supp1 Supplementary data

Patients were followed up until gout diagnosis or prescription or self-reported consumption of gout medication, whichever came first. Information on death or migration was obtained from the Cause of Death Register and the Register of the Total Population.^33^ The cut-off date for analyses was 31 December 2013. The median follow-up was 18.6 (range: 0–26) years for the control group and 18.8 (range: 0–26) years for the surgery group.

As there is no exact definition of hyperuricaemia,^34^ we chose to use serum uric acid levels of 6.8 mg/dL as a cut-off, since this is the saturation point for uric acid in physiological conditions.^4^ In a subgroup analysis on hyperuricaemia incidence at follow-up, only subjects with serum uric acid <6.8 mg/dL at baseline were included (see online supplementary figure S1B). Participants were followed up until diagnosis of hyperuricaemia or their last follow-up examination, whichever occurred first. The cut-off date for analyses was 30 June 2015. Median follow-up was 10 (range: 0–20) years for both groups.

### Statistical analysis

Differences between treatment groups in baseline continuous parameters and changes in body mass index and serum acid uric levels during follow-up were evaluated by linear regression analysis adjusted for sex, baseline age and body mass index. Categorical variables were compared using χ^2^ test.

Time to incident gout or hyperuricaemia was assessed by Kaplan-Meier estimates of cumulative incidence rates and compared between the groups by log-rank test. HRs for the risk of gout and hyperuricaemia were estimated using Cox proportional hazard models. HRs are shown unadjusted and adjusted for preselected risk factors. The association between baseline risk factors and the effect of bariatric surgery on the incidence of gout was assessed using a risk factor–treatment interaction analysis. For continuous variables, the interaction test used the original variable. A total of 18 post-hoc subgroup analyses were executed and reported; no adjustment for multiple testing was performed. One out of twenty interaction tests would be expected to be statistically significant due to chance alone. We calculated the number of surgery procedures needed to be performed to prevent one incident gout or hyperuricaemia event over 15 years (number needed to treat, NNT) as the reciprocal of the absolute risk difference (obtained from Kaplan-Meier risk estimates) between subjects from the surgery and control groups.

Two-sided p values <0.05 were considered statistically significant. The intention-to-treat principle was used unless otherwise indicated. In a per-protocol analysis, we censored subjects from the control group who underwent bariatric surgery and those from the surgery group who underwent additional bariatric procedures during follow-up. All statistical analyses were performed with the Statistical Package for Social Science (V.19.0.0; SPSS, Chicago, Illinois, USA).

## Results

### Baseline characteristics and 2-year and 10-year changes in body mass index

Compared with the participants from the control group, surgery group participants were younger and had a worse metabolic profile at baseline, including higher body mass index, waist circumference, blood pressure, blood glucose, serum insulin, total cholesterol and triglycerides, as well as higher prevalence of type 2 diabetes, hypertension and smoking habit (table 1). Serum uric acid levels were similar in both the control and surgery groups. There was no difference in the percentage of subjects with baseline serum uric acid ≥6.8 mg/dL between the treatment groups.

**Table 1:** Baseline characteristics of study participants

Characteristic	Control group (no=1999)	Surgery group (no=1982)	p Value
Age, year	49±6	47±6	<0.001
Sex, no. men (%)	568 (28)	573 (29)	0.73
Body mass index*	40±5	42±4	<0.001
Waist circumference, cm	117±9	121±10	0.005
Systolic blood pressure, mm Hg	138±18	145±19	<0.001
Diastolic blood pressure, mm Hg	85±11	90±11	<0.001
Hypertension, no. (%)	1267 (63)	1545 (78)	<0.001
Uric acid, mg/dL	5.9±1.3	6.0±1.3	0.25
Uric acid ≥6.8 mg/dL, no. (%)	463 (23)	502 (25)	0.11
Creatinine, mg/dL	0.8±0.1	0.8±0.1	0.71
Blood glucose, mg/dL	88±32	94±36	<0.001
Insulin, mU/L	18±11	22±14	<0.001
Diabetes, no. (%)	249 (13)	334 (17)	<0.001
Total cholesterol, mg/dL	217±43	228±43	<0.001
Triglycerides, mg/dL	179±125	200±137	<0.001
Alcohol intake, g/day	5.3±8.1	5.2±7.3	0.76
Previous cardiovascular events, no. (%)	44 (2)	43 (2)	0.95
Smoking, no. (%)	417 (21)	513 (26)	<0.001
Drugs associated with gout risk†, no. (%)	414 (21)	433 (22)	0.38

Plus–minus values are means±SD.

*The body mass index is the weight in kilograms divided by the square of the height in metres.

**†**Drugs associated with gout risk: diuretics, β-blockers, low-dose acetylsalicylic acid, statins, ciclosporin and organic nitrates.

At the 2-year follow-up, body mass index had decreased by about 23% in the surgery group (95% CI −24 to −23), but no change (+0.1%; 95% CI −0.3 to 0.5) was observed in the control group (p<0.001). After 10 years, the change in body mass index from baseline was −17% (95% CI −17 to −16) in the surgery group and +2% (95% CI 1 to 2) in the control group (p<0.001).

### Incidence of gout

**Figure 1:**
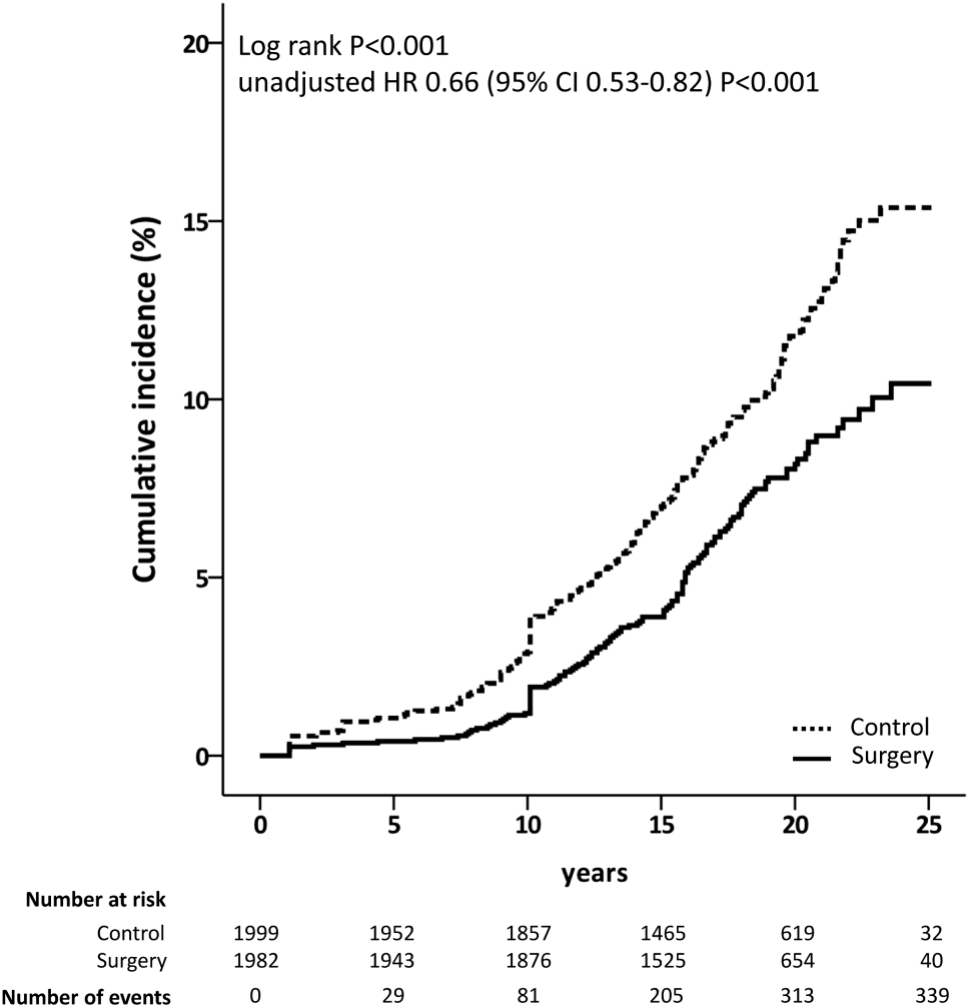
Cumulative incidence of gout. Kaplan-Meier unadjusted estimates of the cumulative incidence of gout in the bariatric surgery group and in the control group.

Bariatric surgery was associated with a lower incidence of gout during a follow-up of up to 26 years (log-rank p<0.001, unadjusted HR 0.66, 95% CI 0.53 to 0.82, p<0.001; figure 1). A total of 201 gout events occurred in the control group compared with 138 events in the surgery group. Similar results were obtained performing a per-protocol analysis (see online supplementary figure S2). All three surgical procedures were associated with a lower incidence of gout compared with the control group (see online supplementary figure S3). In the overall cohort, the difference in absolute risk between groups was 3 percentage points at 15 years, and the NNT by bariatric surgery to prevent one incident gout event was 32 (95% CI 22 to 59).

After multivariable adjustment, bariatric surgery remained associated with a reduced incidence of gout (HR 0.60, 95% CI 0.48 to 0.75, p<0.001; table 2). Age, creatinine levels, uric acid ≥6.8 mg/dL, hypertension, type 2 diabetes, use of medications associated with gout risk and amount of alcohol intake at baseline were associated with an increased risk of developing gout (table 2).

**Table 2:** Adjusted HRs for the incidence of gout

	Adjusted HR (95% CI)	p Value
Surgery vs conventional treatment	0.60 (0.48 to 0.75)	<0.001
Men vs women	0.97 (0.74 to 1.28)	0.82
Age, per 10 years	1.81 (1.49 to 2.19)	<0.001
Body mass index, per 10 kg/m^2^	1.11 (0.87 to 1.40)	0.41
Creatinine, per 1 mg/dL	5.95 (2.28 to 15.5)	<0.001
Uric acid ≥6.8 mg/dL, yes vs no	3.67 (2.89 to 4.64)	<0.001
Hypertension, yes vs no	1.43 (1.03 to 1.97)	0.03
Smoking, yes vs no	0.96 (0.72 to 1.27)	0.78
Type 2 diabetes, yes vs no	1.56 (1.19 to 2.05)	0.001
Triglycerides, per 100 mg/dL	1.02 (0.96 to 1.09)	0.51
Previous cardiovascular events, yes vs no	0.90 (0.49 to 1.67)	0.74
Drugs associated with gout risk, yes vs no	1.39 (1.08 to 1.78)	0.01
Alcohol intake, per 1 g/day	1.02 (1.01 to 1.04)	<0.001

A total of 201 events (10%) occurred in the control group, whereas 138 events (7%) occurred in the surgery group during a follow-up for up to 26 years. The adjusted HRs were calculated using a Cox proportional hazards model based on baseline data.

### Risk factor–treatment interaction analysis

No significant interactions between risk factors at baseline and bariatric surgery were found (see online supplementary table S1). We did not detect any significant difference in NNT between subgroups except when the population was stratified according to serum uric acid levels. The subgroup characterised by hyperuricaemia (defined as serum uric acid ≥6.8 mg/dL) had a lower NNT than the subgroup with low uric acid levels (9, 95% CI 6 to 15 vs 100, 95% CI 44 to ∞). A similar difference in NNT was detected when the population was stratified by median serum uric acid level (see online supplementary table S1).

### Changes in serum uric acid levels and incidence of hyperuricaemia

**Figure 2:**
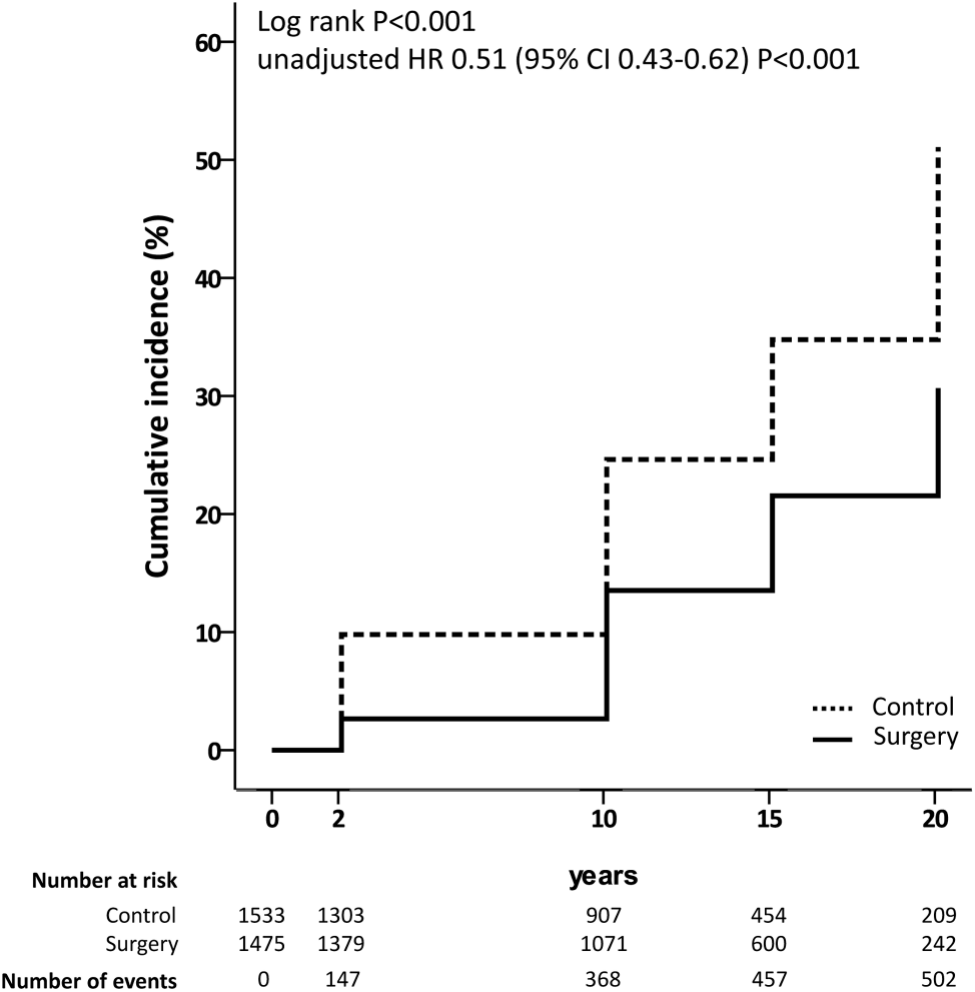
Cumulative incidence of hyperuricaemia. Kaplan-Meier unadjusted estimates of the cumulative incidence of hyperuricaemia in the bariatric surgery and in the control group. Only subjects with serum uric acid <6.8 mg/dL are included in the analysis. Participants were followed up either until the diagnosis of hyperuricaemia or until their last follow-up examination, whichever occurred first.

Serum uric acid levels were significantly lower in the surgery group than in the control group during follow-up (see online supplementary figure S4). After excluding those who were hyperuricemic at baseline, 314 subjects from the control group developed hyperuricaemia during follow-up compared with 188 from the surgery group (log-rank p<0.001, unadjusted HR 0.51, 95% CI 0.43 to 0.62, p<0.001; figure 2). After adjustment, bariatric surgery remained associated with a lower incidence of hyperuricaemia (HR 0.47, 95% CI 0.39 to 0.58, p<0.001; table 3). The difference in absolute risk between groups was 12 percentage points at 15 years, and the estimated NNT to prevent hyperuricaemia was 8 (95% CI 6 to 13).

**Table 3:** Adjusted HRs for the incidence of hyperuricaemia

	Adjusted HR (95% CI)	p Value
Surgery vs conventional treatment	0.47 (0.39 to 0.57)	<0.001
Men vs women	1.48 (1.17 to 1.87)	0.001
Age, per 10 years	1.29 (1.11 to 1.51)	0.001
Body mass index, per 10 kg/m^2^	1.27 (1.03 to 1.55)	0.02
Creatinine, per 1 mg/dL	7.37 (2.68 to 20.3)	<0.001
Hypertension, yes vs no	1.43 (1.13 to 1.81)	0.003
Smoking, yes vs no	0.94 (0.74 to 1.18)	0.58
Type 2 diabetes, yes vs no	1.14 (0.89 to 1.46)	0.30
Triglycerides, per 100 mg/dL	1.06 (0.99 to 1.14)	0.09
Previous cardiovascular events, yes vs no	1.07 (0.58 to 1.96)	0.83
Drugs associated with gout risk, yes vs no	1.55 (1.25 to 1.91)	<0.001
Alcohol intake, per 1 g/day	1.02 (1.00 to 1.03)	0.01

A total of 314 events (21%) occurred in the control group, whereas 188 events (13%) occurred in the surgery group during a follow-up for up to 26 years. The adjusted HRs were calculated using a Cox proportional hazards model based on baseline data.

## Discussion

This matched prospective controlled study shows that bariatric surgery compared with usual care reduces the long-term incidence of gout and hyperuricaemia. In our cohort of obese subjects with no gout diagnosis at baseline, the overall incidence of gout was 34% lower in the surgery group than in the control group during follow-up for up to 26 years. The difference in absolute risk for the incidence of gout between the two groups was 3 percentage points at 15 years, whereas the number of subjects needed to undergo bariatric surgery to prevent one gout event over 15 years was 32. In comparison, the NNT to prevent one cardiovascular event over 15 years was 50 in the same population.^21^ When the analysis was restricted to subjects with normal serum uric acid levels at baseline, bariatric surgery was associated with a 49% lower incidence of hyperuricaemia up to 20 years follow-up compared with the control group. At 15 years, the difference in absolute risk between the two groups was 12 percentage points.

There are no previous long-term studies that have investigated the effect of bariatric surgery on gout incidence in obese subjects without a previous gout diagnosis. Romero-Talamás *et al*^27^ compared 99 obese subjects who underwent bariatric surgery with 56 obese patients who underwent upper abdominal surgery, all of whom had a previous gout diagnosis. Gouty attacks 1 month after surgery were more common in the bariatric surgery group than in patients undergoing other procedures. However, the number of gouty attacks and uric acid levels were lower in the group who underwent bariatric surgery after the first postoperative month and up to 13 months follow-up.

We found no interactions between baseline risk factors and obesity treatment. The preventive effect of bariatric surgery on the incidence of gout was comparable among subjects with body mass index > or ≤40.8, similar to that previously reported for the incidence of type 2 diabetes, cardiovascular disease and cancer.^19^ ^21^ ^22^ This supports the previous findings that criteria other than pure body mass index should be used to select subjects suitable for bariatric surgery.^35^ ^36^ Although no subgroup–treatment interaction was observed when patients were stratified by uric acid level, the NNT to prevent one incidental gout event in subjects with hyperuricaemia at baseline was lower than the NNT in those with baseline low uric acid levels. This result is merely a reflection of the higher risk of developing gout in subjects with hyperuricaemia.

We previously showed that bariatric surgery is associated with lower uric acid levels at 2-year and 10-year follow-up compared with usual care.^17^ Here, we show that the reduction in uric acid levels following bariatric surgery is maintained for up to 20 years. Furthermore, our results show that bariatric surgery prevents development of hyperuricaemia in subjects with normal serum uric acid levels at baseline. The mechanisms behind the reduction in serum uric acid levels following bariatric surgery include among others the improvement in renal function^25^ and insulin resistance,^37^ the reduction in serum triglyceride levels^38^ and possibly even diet changes.^11^ In an uncontrolled study, Dalbeth *et al*^26^ showed that bariatric surgery leads to a reduced inflammatory response to monosodium urate crystals, which may partially contribute to the reduced gout incidence in the surgery group. However, since hyperuricaemia is the main risk factor for gout development,^5^ the decrease in serum uric acid and the prevention of hyperuricaemia are most likely the main factors explaining the association between bariatric surgery and lower risk for gout in obese subjects.

Our study has some limitations. Gout incidence was not a predefined endpoint in the SOS study design. Moreover, we did not include primary care in our analyses, since a complete record of primary care visits is not currently available in Sweden; as gout is often diagnosed and treated in primary care centres, we may therefore have missed some gout diagnoses. For our outcome definition, we used data from the Prescribed Drug Register which also includes information from primary care as well as self-reported data on consumption of allopurinol and colchicine. However, it is important to point out that the register did not start before 2005 and that self-reported drug consumption can be incomplete; moreover, allopurinol and colchicine in Sweden may be prescribed for other indications (tumour lysis syndrome for allopurinol and periodic fevers for colchicine) which are however rare compared with gout. In addition, treatment of acute gout attacks with steroids or non-steroidal anti-inflammatory drugs was not taken into account. Another limitation of the study is that the gold standard for gout diagnosis (detection of monosodium urate crystals in the joint fluid) is rarely used in clinical practice, thus leading to possible false-positive diagnoses in our cohort.^39–41^ However, all these limitations apply equally to both the control and surgery groups. It should be also noted that about 70% of participants of the SOS study are women; since gout is more common in men, this may limit the possibility of generalising our findings.

In conclusion, in a well-characterised and large cohort of obese subjects with long-term follow-up, we have shown that bariatric surgery is associated with a reduced incidence of gout and hyperuricaemia compared with usual care. These results confirm once more that the beneficial effects of bariatric surgery are not limited to weight loss and that they also extend to the prevention of hyperuricaemia and gout.
