# Supplementary material for: Effects of bariatric surgery on gout incidence in the Swedish Obese Subjects study: a non-randomised, prospective, controlled intervention trial
Source: Ann Rheum Dis. 2016 Oct 8;76(4):688–93. doi: 10.1136/annrheumdis-2016-209958 (PMC5530073; doi:10.1136/annrheumdis-2016-209958)
Supplement: Supplementary data [file annrheumdis-2016-209958supp001.pdf]

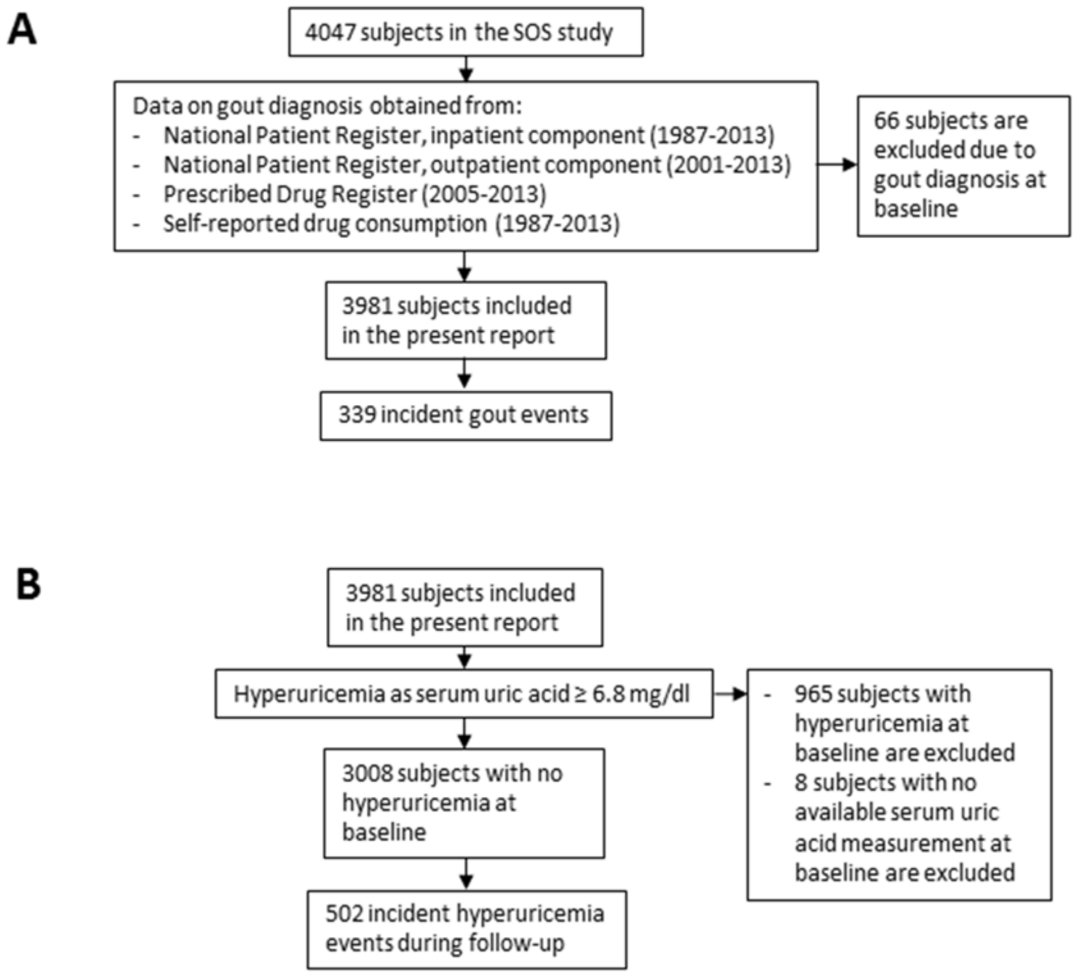


**Supplementary Figure 1. Flow scheme of the present report.** A, analysis on gout incidence. B, analysis on hyperuricemia incidence. Boxes on the right indicate subjects excluded from the present report.

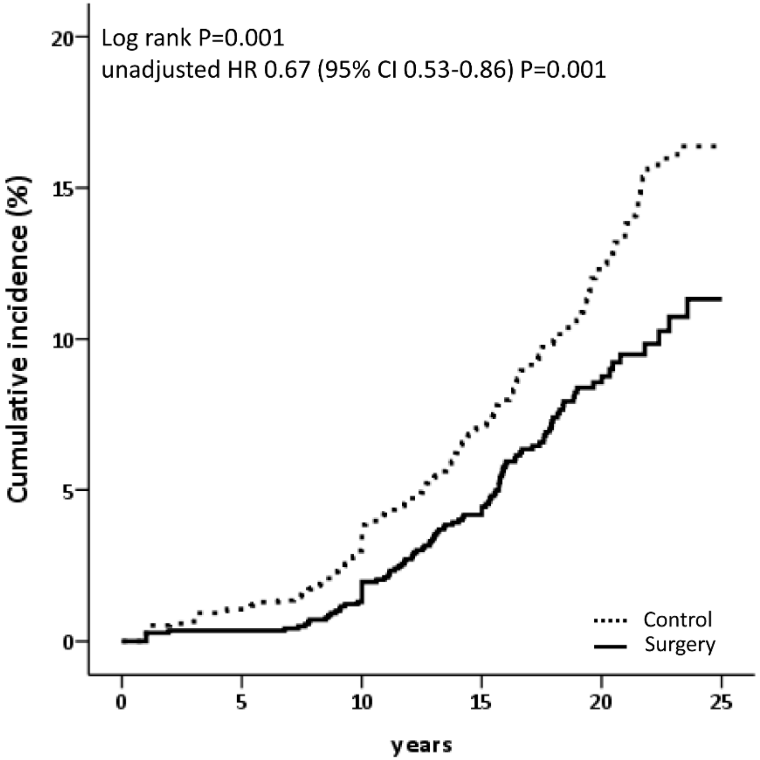


| | | | | | | |
|---|---|---|---|---|---|---|
| **Number at risk** | | | | | | |
| Control | 1715 | 1671 | 1582 | 1257 | 535 | 28 |
| Surgery | 1426 | 1394 | 1341 | 1086 | 456 | 19 |
| **Number of events** | 0 | 23 | 69 | 170 | 261 | 285 |

**Supplementary Figure 2. Per Protocol Analysis of Gout Incidence.** Kaplan-Meier unadjusted estimates of the cumulative incidence of gout in the bariatric surgery group and in the control group. The analyses were performed after excluding subjects from the control group who underwent bariatric surgery as well as subjects from the surgery group who underwent additional bariatric procedures during follow-up. A total of 181 events occurred in the control group (11%, median follow-up 18.7 years [range 0-26]) as compared to 104 in the surgery group (7%, median follow-up 18.7 years [range 0-26]). Survival distributions in the control and surgery groups were compared by a log rank test. After adjustment (for sex, baseline age, body-mass index, serum creatinine, smoking habit, hypertension, type 2 diabetes, hyperuricemia, previous cardiovascular events, self-reported co-medications associated with gout risk and alcohol intake) results are virtually unchanged: HR 0.60 (95% CI 0.46 to 0.77), P<0.001. The estimated number needed to treat (NNT) with bariatric surgery to prevent one event over the course of 15 years was 35 (95% CI 23 to 73).

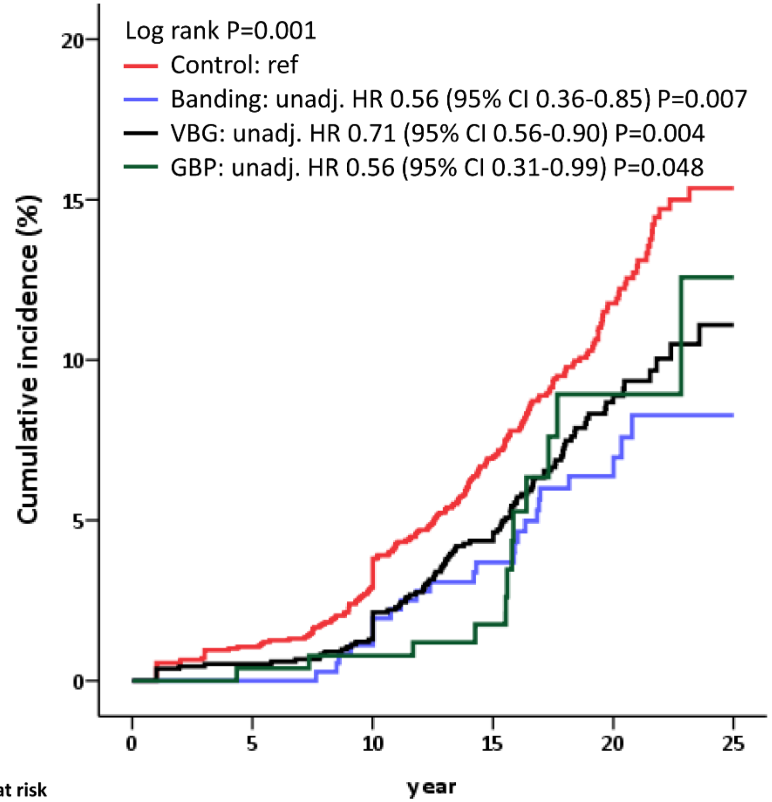


| Number at risk | | | | | | |
|---|---|---|---|---|---|---|
| Control | 1999 | 1952 | 1857 | 1465 | 619 | 32 |
| Banding | 372 | 362 | 355 | 302 | 159 | 11 |
| VBG | 1347 | 1319 | 1278 | 1087 | 455 | 23 |
| GBP | 263 | 257 | 243 | 136 | 40 | 6 |
| **Number of events** | 0 | 29 | 81 | 205 | 313 | 339 |

**Supplementary Figure 3. Cumulative Incidence of Gout Stratified by Surgical Procedure.** Kaplan-Meier unadjusted estimates of the cumulative incidence of gout in the bariatric surgery group and in the control group according to intention-to-treat principle. A total of 201 events occurred in the control group (10%, median follow-up 18.6 years [range 0-26]) as compared to 24 in the banding group (6%, median follow-up 19.6 years [range 0-26]), 102 in the vertical banded gastroplasty (VBG) group (8%, median follow-up 18.9 years [range 0-26]), and 12 in the gastric bypass (GBP) group (5%, median follow-up 15.1 years [range 0-26]). Survival distributions in the control and surgery groups were compared by a log rank test. After adjustment (for sex, baseline age, body-mass index, serum creatinine, smoking habit, hypertension, type 2 diabetes, previous cardiovascular events and self-reported co-medications associated with gout risk) the hazard ratios were as follows: banding, HR 0.52 (95% CI 0.34 to 0.80) P=0.003; VBG, HR 0.66 (95% CI 0.51 to 0.85) P=0.001; GBP, HR 0.50 (95% CI 0.28

to 0.90) P=0.02. The estimated number needed to treat (NNT) with bariatric surgery to prevent one event over the course of 15 years was 31 (95% CI 21 to 60) for banding, 39 (95% CI 24 to 107) for VBG and 19 (95% CI 14 to 33) for GBP.

**Supplementary Table 1. Risk Factor-Treatment Interaction Analyses for Gout Incidence.**

| Variable, sub-grouping at baseline | Incidence of Gout, IR per 1000 Person-Years* | | | | | | Relative Treatment Effects | | | |
|---|---|---|---|---|---|---|---|---|---|---|
| | Surgery | | | Control | | | | | | |
| | No. | Person-Years | IR (95%CI) | No. | Person-Years | IR (95%CI) | Hazard Ratio (95% CI) | P Value for HR | P Value for inter-action† | NNT (95% CI) |
| **Total** | | | | | | | 0.66 (0.53-0.82) | <0.001 | - | 32 (22-59) |
| **Age, years** | | | | | | | | | | |
| ≤47.7 | 1093 | 19925 | 2.4 (1.8-3.1) | 902 | 16414 | 3.8 (3.0-4.9) | 0.61 (0.42-0.89) | 0.01 | 0.33 | 53 (28-467) |
| >47.7 | 889 | 15537 | 5.9 (4.8-7.2) | 1097 | 18337 | 7.5 (6.4-8.9) | 0.76 (0.58-0.98) | 0.04 | | 28 (18-81) |
| **Sex** | | | | | | | | | | |
| Men | 573 | 9871 | 5.7 (4.4-7.4) | 568 | 9511 | 9.4 (7.6-11.5) | 0.60 (0.43-0.84) | 0.003 | 0.47 | 17 (10-44) |
| Women | 1409 | 25591 | 3.2 (2.6-4.0) | 1431 | 25240 | 4.4 (3.7-5.3) | 0.70 (0.53-0.93) | 0.01 | | 50 (29-170) |
| **Body-mass index** | | | | | | | | | | |
| ≤40.8 | 787 | 14165 | 3.2 (2.-4.3) | 1213 | 21342 | 5.5 (4.6-6.6) | 0.58 (0.41-0.82) | 0.002 | 0.44 | 32 (20-86) |
| >40.8 | 1195 | 21297 | 4.3 (3.5-5.3) | 786 | 13409 | 6.2 (5.0-7.7) | 0.67 (0.50-0.90) | 0.008 | | 29 (18-90) |
| **Weight, kg** | | | | | | | | | | |
| ≤116 | 874 | 15893 | 2.6 (2.0-3.6) | 1149 | 20300 | 5.3 (4.4-6.4) | 0.49 (0.34-0.70) | <0.001 | 0.98 | 25 (18-43) |
| >116 | 1108 | 19570 | 4.9 (4.0-6.0) | 850 | 14451 | 6.4 (5.3-7.9) | 0.74 (0.56-0.99) | 0.04 | | 36 (19-245) |
| **Waist, cm** | | | | | | | | | | |
| ≤118 | 896 | 16472 | 2.3 (1.7-3.2) | 1196 | 21177 | 5.0 (4.1-6.1) | 0.46 (0.32-0.66) | <0.001 | 0.21 | 27 (19-50) |
| >118 | 1081 | 18907 | 5.2 (4.3-6.3) | 803 | 13573 | 7.0 (5.7-8.6) | 0.72 (0.55-0.96) | 0.02 | | 30 (18-110) |
| **Systolic blood pressure, mmHg** | | | | | | | | | | |
| ≤140 | 1002 | 17908 | 2.5 (1.8-3.3) | 1284 | 22551 | 4.6 (3.8-5.5) | 0.53 (0.37-0.75) | <0.001 | 0.19 | 43 (26-130) |
| >140 | 975 | 17477 | 5.3 (4.3-6.5) | 711 | 12122 | 8.0 (6.5-9.7) | 0.64 (0.48-0.85) | 0.002 | | 19 (12-39) |
| **Diastolic blood pressure, mmHg** | | | | | | | | | | |
| ≤88 | 825 | 14568 | 2.5 (1.8-3.4) | 1208 | 20858 | 4.8 (4.0-5.9) | 0.50 (0.34-0.73) | <0.001 | 0.11 | 29 (19-69) |
| >88 | 1151 | 20790 | 4.9 (4.0-5.9) | 784 | 13764 | 7.2 (5.9-8.8) | 0.66 (0.50-0.87) | 0.003 | | 26 (16-74) |
| **Hypertension** | | | | | | | | | | |
| No | 437 | 7779 | 1.3 (0.7-2.4) | 732 | 12928 | 3.4 (2.5-4.6) | 0.37 (0.19-0.74) | 0.005 | 0.16 | 33 (20-96) |

| | | | | | | | | | | |
|---|---|---|---|---|---|---|---|---|---|---|
| Yes | 1545 | 27684 | 4.6 (3.9-5.5) | 1267 | 21823 | 7.2 (6.2-8.4) | 0.63 (0.50-0.79) | <0.001 | | 25 (17-49) |
| **Uric acid, mg/dl** | | | | | | | | | | |
| ≤5.9 | 951 | 17149 | 1.7 (1.2-2.4) | 1062 | 18991 | 2.5 (1.9-3.4) | 0.66 (0.41-1.04) | 0.07 | 0.67 | 333 (59-∞)†† |
| >5.9 | 1026 | 18229 | 5.9 (4.9-7.1) | 934 | 15726 | 9.7 (8.3-11.4) | 0.59 (0.46-0.75) | <0.001 | | 15 (11-23) |
| **Uric acid ≥ 6.8 mg/dl** | | | | | | | | | | |
| No | 1475 | 26610 | 2.2 (1.7-2.8) | 1533 | 27394 | 3.2 (2.6-4.0) | 0.67 (0.48-0.93) | 0.02 | 0.32 | 100 (44-∞)†† |
| Yes | 502 | 8768 | 8.9 (7.1-11.1) | 463 | 7323 | 15.4 (12.8-18.6) | 0.56 (0.42-0.74) | <0.001 | | 9 (6-15) |
| **Creatinine, mg/dl** | | | | | | | | | | |
| ≤0.78 | 1048 | 19220 | 3.3 (2.6-4.3) | 1017 | 18442 | 4.3 (3.4-5.3) | 0.77 (0.55-1.07) | 0.11 | 0.55 | 48 (26-242) |
| >0.78 | 930 | 16180 | 4.4 (3.5-5.6) | 980 | 16281 | 7.5 (6.3-8.9) | 0.58 (0.43-0.77) | <0.001 | | 24 (15-55) |
| **Insulin, mU/l** | | | | | | | | | | |
| ≤16.9 | 844 | 15349 | 2.8 (2.1-3.8) | 1135 | 19958 | 5.1 (4.2-6.2) | 0.54 (0.38-0.77) | 0.001 | 0.99 | 29 (20-58) |
| >16.9 | 1129 | 19967 | 4.7 (3.8-5.8) | 861 | 14758 | 6.8 (5.6-8.2) | 0.68 (0.51-0.90) | 0.008 | | 26 (16-66) |
| **Type 2 diabetes** | | | | | | | | | | |
| No | 1641 | 29682 | 3.6 (3.0-4.3) | 1746 | 29681 | 5.1 (4.3-5.9) | 0.69 (0.54-0.89) | 0.003 | 0.15 | 37 (24-86) |
| Yes | 334 | 5675 | 5.6 (4.0-8.0) | 249 | 3905 | 11.3 (8.4-15-1) | 0.48 (0.30-0.75) | 0.001 | | 14 (8-62) |
| **Total cholesterol, mg/dl** | | | | | | | | | | |
| ≤219 | 891 | 15769 | 3.4 (2.6-4.4) | 1100 | 19098 | 4.5 (3.6-5.6) | 0.73 (0.52-1.03) | 0.07 | 0.29 | 34 (21-91) |
| >219 | 1087 | 19631 | 4.2 (3.4-5.2) | 897 | 15625 | 7.4 (6.1-8.8) | 0.57 (0.43-0.75) | <0.001 | | 26 (16-62) |
| **Alcohol intake, g/day** | | | | | | | | | | |
| ≤2.15 | 976 | 17775 | 3.2 (2.5-4.2) | 1014 | 17886 | 4.6 (3.7-5.8) | 0.67 (0.48-0.94) | 0.02 | 0.81 | 45 (25-254) |
| >2.15 | 1006 | 17688 | 4.6 (3.7-5.7) | 984 | 16847 | 7.0 (5.9-8.4) | 0.64 (0.48-0.85) | 0.002 | | 23 (15-48) |
| **Previous cardiovascular events** | | | | | | | | | | |
| No | 1939 | 34792 | 3.9 (3.3-4.6) | 1955 | 34103 | 5.7 (4.9-6.5) | 0.67 (0.54-0.84) | <0.001 | 0.32 | 32 (22-59) |
| Yes | 43 | 670 | 4.5 (1.4-13.9) | 44 | 648 | 12.3 (6.2-24.7) | 0.35 (0.09-1.34) | 0.13 | | 31 (6-∞) |
| **Smoking** | | | | | | | | | | |
| No | 1467 | 26430 | 4.3 (3.6-5.2) | 1572 | 27537 | 5.7 (4.9-6.7) | 0.74 (0.58-0.94) | 0.01 | 0.10 | 36 (22-90) |
| Yes | 513 | 8999 | 2.7 (1.8-4.0) | 417 | 7076 | 5.7 (4.1-7.7) | 0.46 (0.28-0.77) | 0.003 | | 27 (15-141) |

| | | | | | | | | | | | |
|---|---|---|---|---|---|---|---|---|---|---|---|
| **Drugs associated with gout risk†††** | | | | | | | | | | | |
| | No | 1549 | 27816 | 2.9 (2.4-3.7) | 1585 | 27935 | 4.5 (3.8-5.4) | 0.64 (0.49-0.85) | 0.002 | 0.97 | 40 (26-92) |
| | Yes | 433 | 7646 | 7.3 (5.6-9.5) | 414 | 6916 | 11.0 (8.8-13.8) | 0.64 (0.45-0.91) | 0.01 | | 17 (10-69) |

Risk factor-treatment interaction for gout diagnosis in subgroups and number needed to treat (NNT) by bariatric surgery to prevent one gout event over 15 years. To convert the values for uric acid to micromoles per liter, multiply by 59.48. To convert the values for creatinine to micromoles per liter, multiply by 88.4. To convert the values for glucose to millimoles per liter, multiply by 0.05551. To convert the values for insulin to picomoles per liter, multiply by 6.945. To convert the values for cholesterol to millimoles per liter, multiply by 0.02586. CI denotes confidence interval.

* Gout events in high-risk and low-risk subgroups. For continuous variables, subgrouping is based on median baseline values. IR denotes incidence rate.

† For each continuous variable, the test of interaction was calculated using the original continuous variable. Dichotomous variables, including uric acid when dichotomized, could have one of two values (e.g. men/women or uric acid ≥ 6.8 mg/dl, yes/no). The interaction P value reflects the difference in relative treatment effect between the indicated high- and low-risk subgroups.

†† Significant difference between the two subgroups with respect to number needed to treat.

††† Drugs associated with gout risk: diuretics, β-blockers, low-dose acetylsalisylic acid, statins, cyclosporine and organic nitrates.

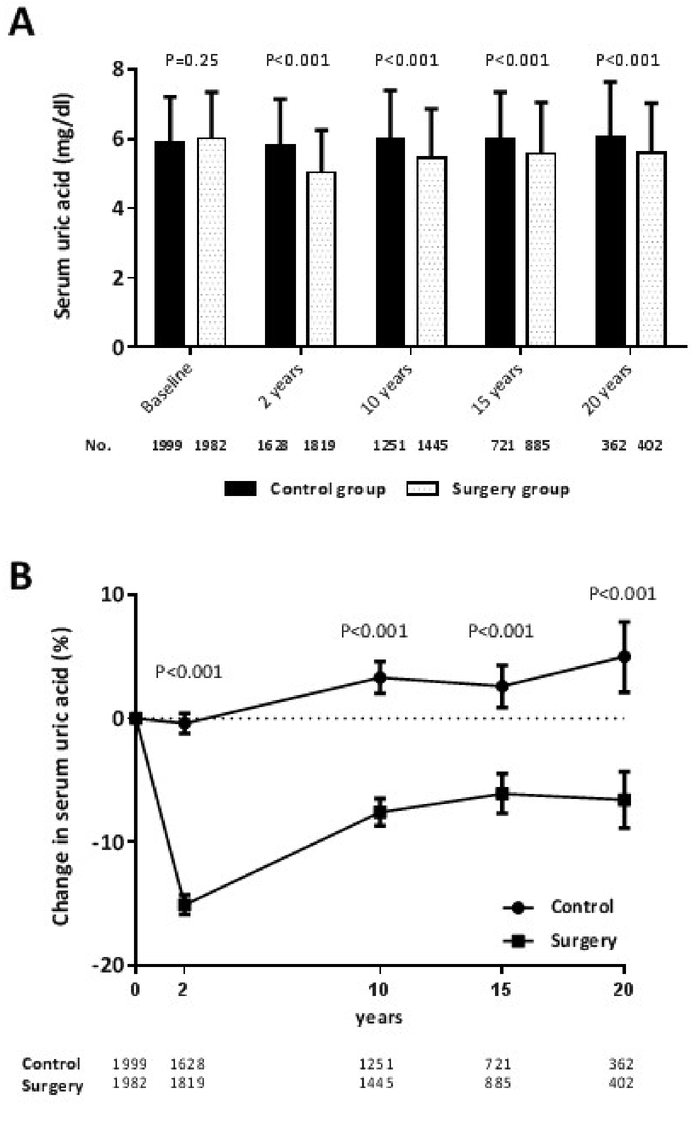


**Supplementary Figure 4. Serum Uric Acid Levels (A) and Changes (B) in the Control and Surgery Group during Follow-Up.** Serum uric acid levels are shown as mean and standard deviation (A). Serum uric acid changes are shown as mean and 95% confidence interval (B). P values have been calculated by linear regression analysis adjusted for sex and baseline age and body-mass index. Further adjustment for risk factors does not affect the result.
